# Case Report: Identification of likely recurrent *CEP290* mutation in a child with Joubert syndrome and cerebello-retinal-renal features.

**DOI:** 10.12688/f1000research.109628.2

**Published:** 2023-03-31

**Authors:** Lidvana Spahiu, John A Sayer, Emir Behluli, Thomas Liehr, Gazmend Temaj

**Affiliations:** 1 Pediatric Clinic, University Clinical Center of Kosovo, Prishtina, Kosovo (Serbia and Montenegro); 2 Newcastle Biomedical Research Centre, NIHR, Newcastle upon Tyne, Tyne and Wear, UK; 3 Renal Services, Newcastle upon Tyne Hospitals National Health Service Foundation Trust, Newcastle, Tyne and Wear, NE7 7DN, UK; 4 Translational and Clinical Research Institute, Newcastle University, Newcastle upon Tyne, Tyne and Wear, NE1 3BZ, UK; 5 Institute of Human Genetics, Jena University Hospital, Jena, D-07747, Germany; 6 Faculty of Pharmacy, College UBT, Prishtina, Kosovo (Serbia and Montenegro)

**Keywords:** Joubert syndrome, CEP290, molecular genetics, ciliopathy, retinal dystrophy

## Abstract

**Background. **Joubert syndrome (JS) is a rare autosomal recessive ciliopathy with an estimated prevalence of 1 in 100,000. JS is characterized by hyperpnoea, hypotonia, ataxia, developmental delay and various neuropathological abnormalities in the brain including cerebellar hypoplasia and cerebellar vermis aplasia. JS can also have variable multi-organ involvement, including the retina, kidneys, liver, and musculoskeletal system.

**Methods and Results**. Here we report a clinical description of two-year-old girl presenting with breathing difficulties, hyperechoic kidneys with loss of corticomedullary differentiation. Brain magnetic resonance imaging revealed the typical molar tooth sign consistent with a clinical diagnosis of JS and retinal examination showed severe retinal dystrophy leading to blindness. Molecular genetic analysis using whole exome sequencing and Sanger sequence confirmation demonstrated a homozygous mutation (c.5493delA, p.(A1832fs*19) in *CEP290* which segregated from either parent and was consistent with the multisystem ciliopathy phenotype. This precise variant has been described previously in 2 families from the Kosovar-Albanian region suggesting this allele is a recurrent mutation in this population.

**Conclusions. **Mutations in *CEP290 *lead to multisystem ciliopathy syndromes and molecular genetic diagnostics of such cases allows precise diagnosis, screening of at risk relatives and appropriate management.

## Introduction

Joubert syndrome (JS, MIM 213300) is a rare heterogeneous neurodevelopmental disease, being associated with an autosomal or X-chromosomal recessive inheritance. There are at least 34 genes (OMIM PS213300) associated with different subtypes of JS ( Parisi and Glass 1993). JS can present clinically with different symptoms including hypotonia progressing to ataxia, global development delay, eye movement abnormalities (nystagmus and ocular molar apraxia) and dysregulation of breathing. Other clinical features found in JS patients depends upon other systemic involvement but includes retinal dystrophy, hepatic fibrosis, polycystic kidney disease and polydactyly ( Brancati *et al.* 2010; Parisi and Glass 1993; Romani *et al.* 2013; Sayer *et al.* 2006; Valente *et al.* 2013). The prevalence of JS is approximately 1 in 100,000 of live births worldwide. From the currently known JS genes, *TMEM67* is the most frequent disease causing gene in European and Japanese patients ( Bachmann-Gagescu *et al.* 2015; Kroes *et al.* 2016; Suzuki *et al.* 2016) and is one of the most frequent cause of JS with renal involvement ( Fleming *et al.* 2017). *CEP290* is also a frequent cause of JS associated with kidney phenotypes ( Bachmann-Gagescu *et al.* 2015; Vilboux *et al.* 2017). Here we report a patient from Kosovo with a clinical diagnosis of JS with kidney and retinal involvement in whom we identified a homozygous frameshift mutation in *CEP290.*

## Case presentation

The Institutional Review Board of Department of Paediatrics, Clinical University Center, Kosovo, approved this work. The parents of the patient provided their written informed consent before clinical and laboratory examinations and for publication of the case. Blood from the patient and their healthy parents were obtained to allow DNA extraction and genetic studies. All the procedures performed in the study were in accordance with the Helsinki Declaration.

**Figure 1.**
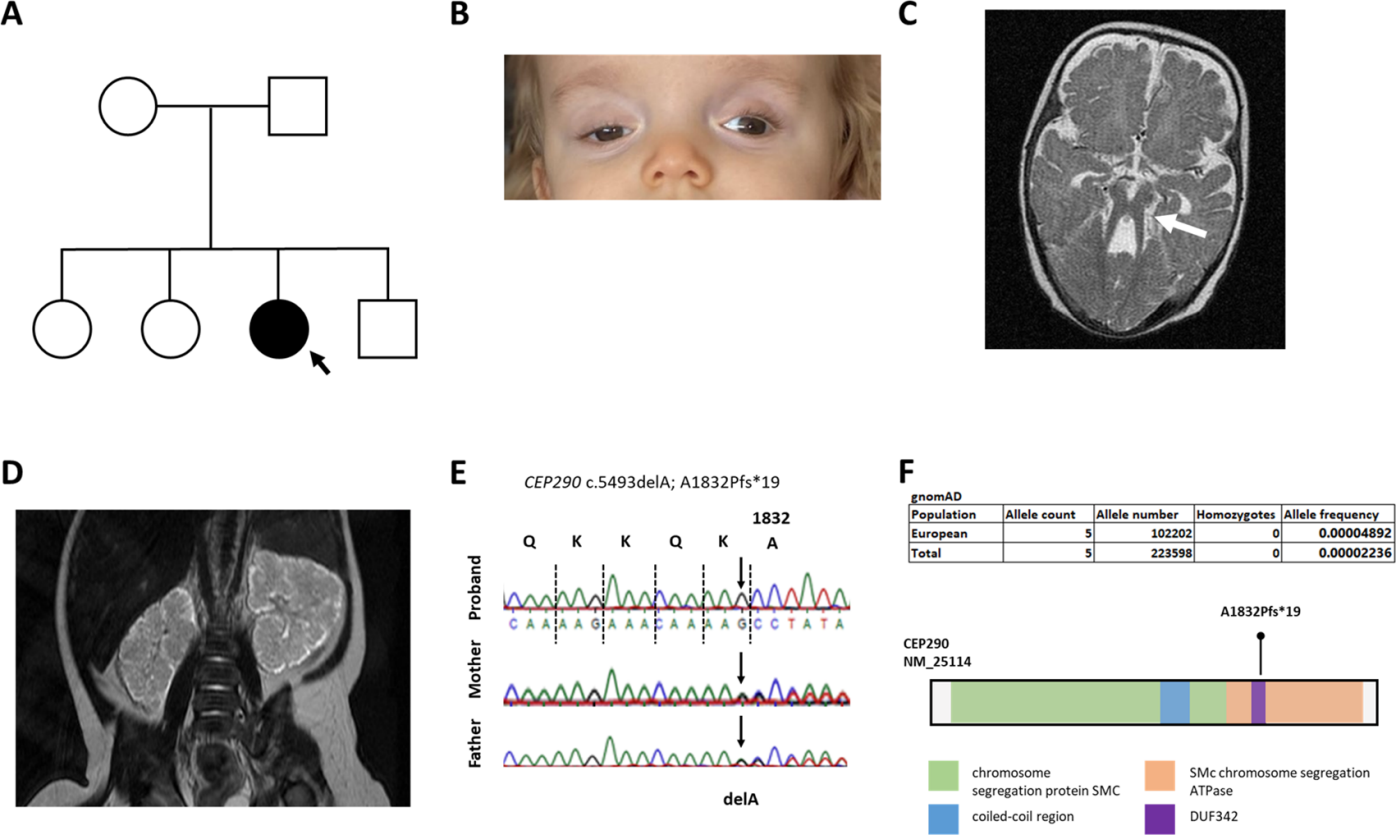
Clinical and molecular genetic features of child with cerebello-retinal-renal phenotype. A. Pedigree diagram; squares, males; circles, females; shaded, affected. B. Photograph of proband aged 3 years, showing right ptosis. C. Brain MRI demonstrating molar tooth sign (arrowed). D. Abdominal MRI demonstrating bilateral enlarged kidneys. E. Sanger sequencing showing homozygous pathogenic frameshift mutation segregating from each parent. F. gnomAD frequencies of pathogenic allele CEP290 NM_25114 c.5493delA; A1832Pfs*19 and CEP290 protein showing domain structure and location of frameshift mutation which truncates C-terminal domains.

The female patient (JBTS-2) was born to a not knowingly consanguineous couple from Kosovo. The patient had three healthy siblings, two older sisters and one younger brother. During antenatal scans, ill-defined brain morphology changes were noted. Following birth, via Caesarean section at 38 weeks gestation, the birth weight was 3460 g, length 50 cm, and head circumference 35 cm. Following birth, the baby became dyspnoeic and cyanotic, and was treated with oxygen therapy (FiO _2_ 30%). Externally, there were no dysmorphic signs. At age of 1 year, the child had evidence of poor growth: weight 7610 g (below 5 ^th^ percentile), height 75 cm (below 25 ^th^ percentile), head circumference 48 cm (below 5 ^th^ percentile). At 2 years of age, the child demonstrated features of developmental delay and was unable to sit or walk. She had started mumbling at the age of 18 months but did not develop any coherent speech. She had horizontal gaze nystagmus and ptosis in right eye. Abdominal ultrasound scanning showed enlarged, hyperechoic kidneys with loss of corticomedullary differentiation, with a number of small cysts up to 12 mm in diameter. There was evidence of progressive chronic kidney disease with serum creatinine 243 μmol/L. Magnetic resonance imaging (MRI) of the head and neck suggested a Dandy Walker anomaly and a typical “molar tooth sign”. Eye examination revealed severe retinal dystrophy and ptosis of the right eyelid ( Table 1). Molecular genetic analysis using exome sequencing and Sanger sequence confirmation revealed a homozygous frameshift mutations c.5493delA; p.A1832fs*19 in *CEP290* ( Figure 1). This variant segregated from each parent.

**Table 1.** Clinical features in presenting case.

Neurological	Retinal	Renal
Developmental delay Nystagmus, right eye ptosis Dandy Walker anomaly Molar tooth sign	Retinal dystrophy	Enlarged hyperechoic kidneys Loss of corticomedullary differentiation Cystic kidneys

## Discussion

JS originally was described in 1968 in a Canadian French family an index patient showing malformation of the cerebellar vermis, with intellectual disability, ataxia, abnormal eye movements, and episodic hyperpnoea ( Brancati *et al.* 2010; Joubert *et al.* 1969). JS has several phenotypic subtypes including “pure” JS, JS with ocular defects, JS with renal defects, JS oculorenal disorders, JS with hepatic disorders and JS with orofaciodigital defects. Of the known 34 genes implicated in JS, 33 genes are autosomal recessive, and one is X-linked ( Coene *et al.* 2009; Parisi 2009). The most striking clinical features for JS are episodic hyperpnoea and apnoea during the neonatal period, ocular disorders, hypotonia truncal ataxia, developmental delay and intellectual impairment ( Kumar *et al.* 2007; Poretti *et al.* 2011).

Biallelic variants in *CEP290* were originally described in families with Joubert syndrome with cerebello-retinal- renal phenotypes ( Sayer *et al.* 2006). Subsequently, *CEP290* mutations have been associated with a wide phenotypic spectrum that includes Leber congenital amaurosis, Senior Løken syndrome, Joubert syndrome, Meckel syndrome and Bardet Biedl syndrome ( Valente *et al.* 2013). Despite the identification and reporting of large numbers of *CEP290* pathogenic alleles, mutations, no clear genotype-phenotype correlations have been established. Therefore the ability to predictive preicise phenotypes and outcomes of patients with CEP290 pathogenic alleles remains limited ( Coppieters *et al.* 2010). Even with two loss of function alleles the phenotype can be limited to Leber congenital amaurosis in some cases and lead to JS in others ( Perrault *et al.* 2007).

Here we report a patient with a molar tooth sign on brain MRI imaging with retinal and kidney involvement in whom we found using exome sequencing a homozygous frameshift mutation in *CEP290* (NM_025114.4 c.5493delA; p.A1832fs*19, ClinVar https://www.ncbi.nlm.nih.gov/clinvar/variation/56739/). This variant, located in exon 29 and predicted to truncate the C-terminus of the CEP290 protein ( Figure 1), has been identified in a wide spectrum of ciliopathy phenotypes from Leber congenital amaurosis ( Carss *et al.* 2017; Cideciyan *et al.* 2011; Coppieters *et al.* 2010; Feldhaus *et al.* 2020), JS with renal and retinal involvement ( Travaglini *et al.* 2009) and Meckel syndrome ( Frank *et al.* 2008; Tallila *et al.* 2009) ( Table 2). Interestingly, of these families with Meckel syndrome and the *CEP290* c.5493delA allele, one consanguineous multiplex family and a second consanguineous family, not knowingly related to the first, were from the Kosovo-Albanian region suggesting this allele might be a recurrent or founder allele from this population. The lack of genotype-phenotype even with this exact variant in families from the same geographical region is noteworthy. The previously reported cases with the homozygous c.5493delA allele in *CEP290* had a classical Meckel syndrome phenotype whilst the case we present here is typical for a multisystem JS. Clearly, there may be modifier alleles or unknown factors influencing the phenotype, and *CEP290* provides a good example of a gene where genotype-phenotype correlations are absent ( Coppieters *et al.* 2010). There is some evidence of a modifier allele for the kidney phenotype relating to *CEP290* mutations ( Ramsbottom *et al.* 2020), but the neurological variability remains unexplained.

**Table 2.** Spectrum of phenotypes seen with *CEP290* allele c.5493delA; A1832Pfs*19.

Case ID	Clinical phenotype	Second *CEP290* allele	Reference
JBTS-2	Joubert syndrome	Homozygous c.5493delA	This report
CEP290_21	Leber congenital amaurosis	Compound heterozygous with c.5587-1G>C	( Feldhaus *et al.* 2020)
G001347	Retinal dystrophy	N/A	( Carss *et al.* 2017)
P8	Leber congenital amaurosis	Compound heterozygous with c.2991+1655A>G	( Cideciyan *et al.* 2011)
LCA-16	Leber congenital amaurosis	Compound heterozygous with p.Cys998*	( Coppieters *et al.* 2010)
COR001	Joubert syndrome	Compound heterozygous with del ex 42-54	( Travaglini *et al.* 2009)
N/A - 2 families	Meckel syndrome	Compound heterozygous with 5489delA	( Tallila *et al.* 2009)
Index family and Family 972	Meckel syndrome ^$^	Homozygous c.5493delA	( Frank *et al.* 2008)

^$^ Two consanguineous families of Kosovar-Albanian origin. Fetuses from the index family had classical features of MKS including grossly enlarged cystic dysplastic kidneys, hepatobiliary ductal plate malformation, postaxial polydactyly, and occipital meningoencephalocele. Fetuses from Family 972 had cystic dysplastic kidneys, postaxial hexadactyly of the left hand, microcephaly with anoccipital meningoencephalocele and hepatobiliary ductal plate malformation was present in the first affected fetus.

In conclusion, JS is often a multi-organ primary ciliopathy syndrome and patients should undergo a clinical and imaging diagnostic protocol to evaluate possible different abnormalities ( Bachmann-Gagescu *et al.* 2020) and genetic testing to allow identification of the underlying cause. Our results highlight the importance of combining clinical and radiological features of JS with molecular genetic analysis to allow a precise diagnosis of JS. An accurate diagnosis helps in genetic counselling, early management of genetic disorders and offers prenatal diagnosis as an option for future pregnancies. It also allows genotype-phenotype correlations to be determined and allows new information to be gained regarding population specific disease alleles.

### Data availability statement

The original contributions presented in the study are publicly available on LOVD ( Fokkema *et al.* 2011). This data can be found here: https://databases.lovd.nl/shared/individuals/00402012.

Data are available under the terms of the Creative Commons Zero “No rights reserved” data waiver (CC0 1.0 Public domain dedication).

## Ethics statement

The Institutional Review Board of Department of Paediatrics, Clinical University Center, Kosovo, approved this work. The studies involving human participants were reviewed and approved by North-East Newcastle and North Tyneside 1 Research Ethics Committee (18/NE/350). Written informed consent to participate in this study was provided by the participants’ legal guardian/next of kin. Written informed consent was obtained from the minor(s)’ legal guardian/next of kin for the publication of any potentially identifiable images or data included in this article.

## Author contributions

The study was conceived and designed by JS. JS, LS, EB, TL and GT contributed to the acquisition, analysis of data, and writing the first draft. LS contributed to collecting the data and communicated with the patient’s family. JS contributed to molecular genetic studies and *in silico* analysis. The final version was edited by JS. All authors edited and approved the final manuscript.
