# Supplementary material for: Case Report: Identification of likely recurrent CEP290 mutation in a child with Joubert syndrome and cerebello-retinal-renal features
Source: F1000Res. 2023 Mar 31;11:388. Originally published 2022 Apr 4. [Version 2] doi: 10.12688/f1000research.109628.2 (PMC10196526; doi:10.12688/f1000research.109628.2)
Supplement: Peer review history

# Peer review: Reviewer response for version 2

**DOI:** 10.5256/f1000research.145997.r168363

**Published:** 2023-05-18

**Authors:** Muhammad Ansar

**Affiliations:** 1 Department of Biochemistry, Faculty of Biological Sciences, Quaid-i-Azam University, Islamabad, Pakistan

Authors have revised the manuscript as per comments raised during first review. Therefore It can be accepted for indexing.

Are enough details provided of any physical examination and diagnostic tests, treatment given and outcomes?

Yes

Is the case presented with sufficient detail to be useful for other practitioners?

Yes

Is sufficient discussion included of the importance of the findings and their relevance to future understanding of disease processes, diagnosis or treatment?

Partly

Is the background of the case’s history and progression described in sufficient detail?

Yes

Reviewer Expertise:

Human Genetics

I confirm that I have read this submission and believe that I have an appropriate level of expertise to confirm that it is of an acceptable scientific standard.

# Peer review: Reviewer response for version 1

**DOI:** 10.5256/f1000research.121150.r156994

**Published:** 2022-12-14

**Authors:** Karen I. Lange

**Affiliations:** 1 School of Biomolecular and Biomedical Science, University College Dublin, Dublin, Ireland

This case study describes a two-year-old girl with clinical phenotypes that affect her eyes, kidneys, and brain. The “molar tooth sign” was observed on MRI and she was diagnosed with Joubert Syndrome. Exome sequencing revealed she has a homozygous mutation in CEP290(c.5493delA/p.A1832fs*19) that she inherited from both parents. This variant has previously been identified in 2 other families from the Kosovo-Albanian region suggesting it may be a recurrent allele in this population. The clinical description of the patient is thorough and the observation that this may be a founder allele in this population is important. 

 I appreciate that the authors include a table which highlights the spectrum of clinical phenotypes associated with this specific CEP290 variant. It is intriguing that previous reports of individuals that were homozygous for this allele were diagnosed with perinatal lethal Meckel Syndrome while the homozygous patient in this case study is has Joubert Syndrome. It would be worthwhile to add a sentence in the discussion to speculate on why this might be.

 I have some minor recommendations that would help with clarity:

- “ *Blood from the patient and his healthy parents*”  should be her/their.
- “ *no clear signs of foramina magnum obstruction and a typical “molar tooth sign*” This should be reworded because I interpreted this as no MTS was observed (even though it is clearly shown in Figure 1C)
- In Table 1 It would be more clear if the Clinical Phenotype of COR001 was listed as Joubert Syndrome since COR is a subset of Joubert.
- I believe the clinical phenotype of G001347 in Table 1 should be retinal dystrophy not LCA.

Are enough details provided of any physical examination and diagnostic tests, treatment given and outcomes?

Yes

Is the case presented with sufficient detail to be useful for other practitioners?

Yes

Is sufficient discussion included of the importance of the findings and their relevance to future understanding of disease processes, diagnosis or treatment?

Yes

Is the background of the case’s history and progression described in sufficient detail?

Yes

Reviewer Expertise:

Genetics/Ciliopathies

I confirm that I have read this submission and believe that I have an appropriate level of expertise to confirm that it is of an acceptable scientific standard.

# Response

**Published:** 2023-03-15

**Authors:** John Sayer

**Affiliations:** Newcastle University, UK

*I appreciate that the authors include a table which highlights the spectrum of clinical phenotypes associated with this specific CEP290 variant. It is intriguing that previous reports of individuals that were homozygous for this allele were diagnosed with perinatal lethal Meckel Syndrome while the homozygous patient in this case study is has Joubert Syndrome. It would be worthwhile to add a sentence in the discussion to speculate on why this might be.*

*Thank you. We have added a section on genotype phenotype correlation in CEP290.*

*I have some minor recommendations that would help with clarity:*

- *“Blood from the patient and his healthy parents”  should be her/their.*
**Corrected**
- *“no clear signs of foramina magnum obstruction and a typical “molar tooth sign” This should be reworded because I interpreted this as no MTS was observed (even though it is clearly shown in Figure 1C)*
** Reworded**
- *In Table 1 It would be more clear if the Clinical Phenotype of COR001 was listed as Joubert Syndrome since COR is a subset of Joubert.*
**Corrected**
- *I believe the clinical phenotype of G001347 in Table 1 should be retinal dystrophy not LCA.*
**Corrected**

# Peer review: Reviewer response for version 1

**DOI:** 10.5256/f1000research.121150.r140267

**Published:** 2022-08-02

**Authors:** Muhammad Ansar

**Affiliations:** 1 Department of Biochemistry, Faculty of Biological Sciences, Quaid-i-Azam University, Islamabad, Pakistan

In this case report, Spahiu et al. presented a patient with a known mutation in CEP290 gene. Mutations in CEP290 are known to cause Joubert syndrome (JS), Meckel syndrome and Leber congenital amaurosis (LCA). Authors concluded that CEP290 variant is recurrent based on its report in two families sharing common ancestry. The report is well written but discussion can be further improved as specified below;

1. Authors should compare the clinical profile of patients from both families carrying c.5493delA. 
2. It would be nice to add fundus image of this patient.
3. It would be better to compare clinical presentation of patients carrying c.5493delA. variant with other published cases carrying loss of function variants in CEP290 gene.

Are enough details provided of any physical examination and diagnostic tests, treatment given and outcomes?

Yes

Is the case presented with sufficient detail to be useful for other practitioners?

Yes

Is sufficient discussion included of the importance of the findings and their relevance to future understanding of disease processes, diagnosis or treatment?

Partly

Is the background of the case’s history and progression described in sufficient detail?

Yes

Reviewer Expertise:

Human Genetics

I confirm that I have read this submission and believe that I have an appropriate level of expertise to confirm that it is of an acceptable scientific standard, however I have significant reservations, as outlined above.

# Response

**Published:** 2023-03-15

**Authors:** John Sayer

**Affiliations:** Newcastle University, UK

*In this case report, Spahiu et al. presented a patient with a known mutation in CEP290 gene. Mutations in CEP290 are known to cause Joubert syndrome (JS), Meckel syndrome and Leber congenital amaurosis (LCA). Authors concluded that CEP290 variant is recurrent based on its report in two families sharing common ancestry. The report is well written but discussion can be further improved as specified below;*

1. *Authors should compare the clinical profile of patients from both families carrying c.5493delA. *
**Thank you we now provide the clinical profile in a new table 1 of our case with homozygous c.5493delA variant and have added details of our case and additional details for the Kosovar-Albanian cases of MKS to table 2 to allow a direct comparison of features.**
2. *It would be nice to add fundus image of this patient.*
**Unfortunately no fundus image is available**
3. *It would be better to compare clinical presentation of patients carrying c.5493delA. variant with other published cases carrying loss of function variants in CEP290 gene.*
**Thank you, this is a helpful suggestion. We have added some more detailed discussion comparing our case with other cases with loss of function variants in CEP290 gene. There is no real genotype-phenotype with variants in this gene, even with LoF alleles.**
